# Changes in perinatal management and outcomes of extremely preterm infants born below 26 weeks of gestation in a tertiary referral hospital in Sweden: Comparison between 2004–2007 and 2012–2016

**DOI:** 10.1111/aogs.14576

**Published:** 2023-05-22

**Authors:** Emma Aronsson, Malin Holzmann, Marco Bartocci, Ingela Hulthén Varli, Sissel Saltvedt

**Affiliations:** ^1^ Pregnancy Care and Delivery Karolinska University Hospital Stockholm Sweden; ^2^ Department of Women's and Children's Health Karolinska Institutet Stockholm Sweden; ^3^ Department of Neonatology Karolinska University Hospital Stockholm Sweden

**Keywords:** cohort studies, extremely premature infant, infant mortality, perinatal care, preterm birth, survival rates

## Abstract

**Introduction:**

Perinatal management of extremely preterm births in Sweden has changed toward active care from 22–23 gestational weeks during the last decades. However, considerable regional differences exist. This study evaluates how one of the largest perinatal university centers has adapted to a more active care between 2004–2007 and 2012–2016 and if this has influenced infant survival.

**Material and methods:**

In this historical cohort study, women admitted with at least one live fetus and delivered at 22–25 gestational weeks (stillbirths included) at Karolinska University Hospital Solna during April 1, 2004–March 31, 2007, and January 1, 2012–December 31, 2016, were compared regarding rates of obstetric and neonatal interventions, and infant mortality and morbidity. Maternal, pregnancy and infant data from 2004–2007 were obtained from the Extreme Preterm Infants in Sweden Study while data from 2012–2016 were extracted from medical journals and quality registers. The same definitions of interventions and diagnoses were used for both study periods.

**Results:**

A total of 106 women with 118 infants during 2004–2007 and 213 women with 240 infants during 2012–2016 were included. Increases between the study periods were seen regarding cesarean delivery (overall rate 14% [17/118] during 2004–2007 vs. 45% [109/240] during 2012–2016), attendance of a neonatologist at birth (62% [73/118] vs. 85% [205/240]) and surfactant treatment at birth in liveborn infants (60% [45/75] vs. 74% [157/211]). Antepartum stillbirth rate decreased (13% [15/118] vs. 5% [12/240]) and the proportion of live births increased (80% [94/118] vs. 88% [211/240]) while 1‐year survival (64% [60/94] vs. 67% [142/211]) and 1‐year survival without major neonatal morbidity (21% [20/94] vs. 21% [44/211]) among liveborn infants did not change between the study periods. At 22 gestational weeks, interventions rates were still low during 2012–2016, most obvious regarding antenatal steroid treatment (23%), attendance of a neonatologist (51%), and intubation at birth (24%).

**Conclusions:**

Both obstetric and neonatal interventions at births below 26 gestational weeks increased between 2004–2007 and 2012–2016 in this single center study; however, at 22 gestational weeks they were still at a low level during 2012–2016. Despite more infants being born alive, 1‐year survival did not increase between the study periods.

## Graphical abstract

At a Swedish level III university hospital, a more proactive perinatal management has developed. However, after birth at 22–23 gestational weeks, mortality rate remains high.

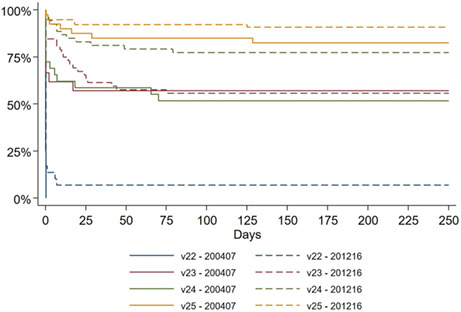

**Abbreviations**
GW: gestational weeks
NICU: neonatal intensity care unit

**Key message**

Management of preterm births at the border of viability remains controversial. At a large level III university hospital, a more active perinatal management at 22–25 gestational weeks led to more infants born alive, however 1‐year survival did not increase.

## 1 INTRODUCTION

Being born extremely preterm is associated with high mortality and morbidity risks, although advances in medical care have led to improvements in survival and long‐term outcomes over the last decades.^1^, ^2^, ^3^, ^4^, ^5^, ^6^ The Swedish EXPRESS study was a national population‐based prospective study of extremely preterm births in Sweden during 2004–2007. One‐year survival of infants born alive at 22 (22 + 0–22 + 6) gestational weeks (GW) was 9.8%, increasing to 85% at 26 GW, clearly higher survival rates than in earlier studies.^7^, ^8^, ^9^, ^10^ The EXPRESS study revealed considerable differences in perinatal management and infant outcomes between regions in Sweden.^11^ Regions with high intensity in perinatal care were found to have lower rates of stillbirth and infant mortality at 1 and 2.5 years of age.^12^ Mortality differences were most pronounced in the subgroups of infants who were born alive but died within 12 h and in those born at 22–24 GW, indicating variations in perinatal management.

Following the results of the EXPRESS study, efforts were made to strengthen and equalize the care of extremely preterm births in Sweden. In 2014, the Swedish National Board of Health and Welfare published general recommendations regarding management of extremely preterm births.^13^ Although a more active perinatal care was applied regionally earlier, it was not until 2016 that national guidelines stated active care to be considered from 22 + 0 GW and recommended from 23 + 0 GW.^1^, ^14^ A follow‐up study of EXPRESS showed that 1‐year survival rate among infants born <28 GW in Sweden improved during a decade, most likely a result of more active perinatal care.^2^

The Stockholm region, including Karolinska University Hospital Solna, had a low perinatal activity score in the EXPRESS study.^11^, ^12^ Based on the EXPRESS findings, management and regional guidelines progressively changed. By comparing management and outcomes of births <26 GW during 2004–2007 with those during 2012–2016, the present study aimed to evaluate whether this hospital has adapted to a more active perinatal approach. We also wanted to explore factors associated with 1‐year survival applying a more active care.

## 2 MATERIAL AND METHODS

This historical cohort study was undertaken at Karolinska University Hospital Solna in Stockholm, Sweden. This is a referral hospital with a level III neonatal intensity care unit (NICU) for women at risk of giving birth extremely preterm in Stockholm, and the only hospital where births before 26 + 0 GW are planned to occur. Births between 22 + 0 and 25 + 6 GW were included. Women were excluded in case of stillbirth at time of admission, or in case of fetal malformation that could affect management and outcome, or if maternal/pregnancy background or infant outcome data were unavailable (eg women not living in Stockholm). As almost all pregnancies (>95%) in Sweden are dated by ultrasound in the first or second trimester or by date of embryo transfer at in vitro fertilization, mode of dating was not registered.

The 2004–2007 study group comprised women and infants participating in the EXPRESS study. EXPRESS included all births at 22 + 0–26 + 6 GW at all obstetric and neonatal departments in Sweden between April 1, 2004, and March 31, 2007. In EXPRESS, data from mothers, deliveries, and infants were prospectively collected and entered in a central database. Morbidity and mortality data were complemented and validated by linking to relevant registers. Missing data were searched for until found or stated to be unavailable. For the current study, relevant data for all births at Karolinska University Hospital Solna at 22 + 0–25 + 6 GW were extracted from the EXPRESS database. Births at 26 GW were not included as they are not exclusively planned to take place at this hospital there is a selection of the most complicated pregnancies to our hospital at this gestation.

The 2012–2016 study group comprised births at 22 + 0–25 + 6 GW at Karolinska University Hospital Solna from January 1, 2012, to December 31, 2016. Maternal, pregnancy and delivery data were obtained from the electronic medical record systems. Data on stillbirths and delivery room deaths were also obtained from the medical records, while data from liveborn infants admitted to NICU were retrieved from the Swedish Neonatal Quality Register (SNQ), which collects data on care and outcomes for all infants admitted to a NICU in Sweden.^15^ If missing in SNQ, data were searched for in the individual medical records. Major morbidity diagnoses were cross‐checked against medical records to ensure accuracy. Obstetric and neonatal interventions were defined in the same way in both study groups. Use of antenatal steroids was defined as any steroids given before birth, and the same applied to the use of tocolytics and antibiotics.

During 2004–2007, regional guidelines recommended transfer to a level III NICU, antenatal steroids from 23 GW, and cesarean delivery and neonatal resuscitation from 24 GW. Updated guidelines 2013 recommended transfer from 22 GW, steroids from 22 + 5 GW, cesarean section to be considered from 23 GW and recommended from 24 GW and neonatal resuscitation from 23 W, in selected cases earlier. Parental information and involvement were emphasized throughout. Magnesium sulfate for neuroprotection was not in use in Sweden during any of the study periods. Delayed cord clamping was sometimes used, but not recommended in guidelines and not documented.

Besides the primary outcome rate of obstetric and neonatal interventions, we also investigated infant outcomes whereof survival at 1 year among liveborn infants was main outcome. One‐year survival without any major morbidity in the neonatal period was a secondary outcome. Major morbidity was defined as one or more of: severe bronchopulmonary dysplasia, retinopathy of prematurity stage >2, intraventricular hemorrhage grade >2, periventricular leukomalacia, or necrotizing enterocolitis. For both study groups, we used the same definitions of all outcome variables, corresponding to those used in the EXPRESS study.^7^ Severe bronchopulmonary dysplasia was defined as need of 30% oxygen for more than 12 h per day at corrected age of 36 + 0 GW; retinopathy of prematurity was defined according to International Classification of retinopathy of prematurity^16^; intraventricular hemorrhage was defined according to Papile et al.^17^; periventricular leukomalacia was defined in accordance with data from de Vries et al.^18^ and necrotizing enterocolitis was defined as at least one clinical symptom (biliary colored gastric residuals, abdominal distention, blood in stool) combined with one or several radiological symptoms (pneumatosis intestinalis, gas in the biliary tract, pneumoperitoneum).^19^ Stillbirth was defined as death of a fetus alive at maternal admission, and classified as antepartum if before, or intrapartum if during labor/cesarean delivery.

### 2.1 Statistical analyses

Results were stratified by gestational week and presented as proportions (percent) and medians (range). Differences in proportions between the study groups were tested by Chi‐square test or Fisher's exact test, while Student's *t*‐test or Wilcoxon rank‐sum test were used for continuous data. When calculating antenatal intervention rates, all infants were included in the denominator, while for neonatal interventions only liveborns. Mortality and survival rates were additionally presented with risk differences (95% confidence interval). A regression analysis was undertaken to explore antenatal factors independently associated to mortality within 1 year among liveborn infants. The 2004–2007 study group was excluded in this analysis as it represented a policy of less active perinatal management and had many missing values in maternal characteristics and neonatal interventions. Included variables were selected based on previous studies (gestational age; maternal age/BMI/hypertensive disorder/preterm premature rupture of the membranes/chorioamnionitis/placental abruption; antenatal steroids/antibiotics/delivery mode/attendance of neonatologist; infant sex/number in birth/Apgar score/weight).^20^, ^21^ We did not perform any statistical test to decide whether to include variables in the final model.

A *p*‐value < 0.05 was considered statistically significant. Data were analyzed using SAS software, version 9.4 (SAS Institute Inc) and STATA version 17.0.

### 2.2 Ethics statement

The 2004–2007 study group was part of the EXPRESS study with ethical approval from the regional research ethics board in Lund, Sweden (42/2004) dated July 1, 2004. The regional ethical review board in Stockholm approved the 2012–2016 part of the study and retrieval of data from EXPRESS (2017/323–31 approved March 8, 2017 and 2020–00710 approved April 7, 2020).

## 3 RESULTS

The overall number of infants born at 22 + 0–25 + 6 GW at Karolinska University Hospital Solna was 131 during 2004–2007 and 289 during 2012–2016. Stillbirths at admission were excluded (12 and 22, respectively), as were infants with limited data on maternal/pregnancy background or infant follow‐up (0 and 25, respectively), and infants with significant malformations (1 with tetralogy of Fallot during 2004–2007, 1 with gastroschisis and 1 with pulmonary stenosis during 2012–2016). A total of 106 women with 118 newborns from 2004–2007 and 213 women with 240 newborns from 2012–2016 remained for analysis.

Median age, median body mass index and the proportion of women aged ≥35 years or with body mass index ≥30 did not differ significantly between the study periods, and neither did the parity (Table 1). Due to missing data for several other background variables in the 2004–2007 study group, comparisons could not be made.

**TABLE 1:** Maternal characteristics of the two study groups. Values are numbers (percentages) unless stated otherwise.

	2004–2007	2012–2016
Number of women	106	213
Maternal age		
Median (range)	33 (15–46)	32 (17–50)
≥35 years	39 (37)	68 (32)
Missing	4 (4)	0
BMI at first antenatal visit		
Median (range)	24 (16–43)	24 (17–40)
≥30	11 (10)	35 (16)
Missing	25 (24)	7 (3)
Country of birth		
Nordic	50 (47)	131 (61)
Non‐Nordic	20 (19)	76 (36)
Missing	21 (34)	6 (3)
Parity		
Nulliparous	68 (64)	130 (61)
Parous	38 (36)	83 (39)
Smoking at first antenatal visit		
Yes	9 (8)	21 (10)
No	76 (72)	181 (85)
Missing	21 (20)	11 (5)
Chronic hypertension		
Yes	2 (2)	6 (3)
No	98 (93)	207 (97)
Missing	6 (6)	0
Prepregnancy diabetes		
Yes	2 (2)	0
No	98 (92)	213 (100)
Missing	6 (6)	0
IVF		
Yes	8 (8)	29 (14)
No	98 (93)	183 (86)
Missing	0	1 (1)
Pre‐eclampsia		
Yes	5 (5)	17 (8)
No	95 (90)	196 (92)
Missing	6 (6)	0
PPROM		
Yes	14 (13)	62 (29)
No	47 (44)	151 (71)
Missing	45 (42)	0
Chorioamnionitis		
Yes	16 (15)	52 (24)
No	84 (79)	161 (76)
Missing	6 (6)	0
Fever^a^		
Yes	8 (8)	23 (11)
No	98 (92)	190 (89)
Placental abruption		
Yes	11 (10)	28 (13)
No	89 (84)	185 (87)
Missing	6 (6)	0
Placenta praevia		
Yes	3 (3)	3 (1)
No	97 (91)	209 (98)
Missing	6 (6)	1 (1)

Abbreviations: BMI, body mass index; IVF, in vitro fertilization; PPROM, preterm premature rupture of membranes.

^a^ Fever >38.0°C measured before delivery.

Antenatal and neonatal interventions are presented in Table 2. From 23 GW, rates of antenatal steroid treatment were high both during 2004–2007 and 2012–2016, while at 22 GW it was low during both periods (12% vs. 23%). There was a more than three‐fold increase in the cesarean delivery rate between the study periods (14% to 45%), seen in all weeks except at 22 GW. During 2012–2016 a neonatologist was present in >90% of births from 23 GW, whereas only in 50% at 22 GW. Due to high number of missing data in the 2004–2007 study groups, comparisons regarding surfactant treatment and intubation must be done with caution.

**TABLE 2:** Obstetric and neonatal interventions, all infants included. Values are numbers (percentages) unless stated otherwise.

	Week 22		Week 23		Week 24		Week 25		Week 22–25		
	2004–2007	2012–2016	2004–2007	2012–2016	2004–2007	2012–2016	2004–2007	2012–2016	2004–2007	2012–2016	*p*‐value*
All infants	9	44	31	57	35	58	43	81	118	240	
Antenatal steroids^a^											
Yes	1 (12)	10 (23)	28 (90)	55 (96)	30 (94)	55 (95)	33 (87)	79 (98)	92 (84)	199 (83)	0.73
No	7 (88)	34 (77)	3 (10)	2 (4)	2 (6)	3 (5)	5 (13)	2 (2)	17 (16)	41 (17)	
Tocolysis^b^											
Yes	5 (71)	20 (53)	20 (67)	36 (80)	22 (71)	35 (80)	25 (86)	41 (80)	72 (74)	132 (74)	0.99
No	2 (29)	18 (47)	10 (33)	9 (20)	9 (29)	9 (20)	4 (14)	10 (20)	25 (26)	46 (26)	
Maternal antibiotics^c^											
Yes	7 (78)	28 (64)	24 (77)	33 (59)	19 (61)	32 (56)	24 (67)	33 (41)	74 (69)	126 (53)	0.005
No	2 (22)	16 (36)	7 (23)	23 (41)	12 (39)	25 (44)	12 (33)	47 (59)	33 (31)	111 (47)	
Cesarean delivery											
Yes	0	1 (2)	0	16 (28)	1 (3)	32 (55)	16 (37)	60 (74)	17 (14)	109 (45)	<0.001
No	0	43 (98)	0	41 (72)	34 (97)	26 (45)	27 (63)	21 (26)	101 (86)	131 (55)	
Neonatologist attending at birth^d^											
Yes	2 (22)	22 (51)	18 (58)	53 (93)	23 (66)	55 (95)	30 (70)	75 (94)	73 (62)	205 (86)	<0.001
No	7 (78)	21 (49)	13 (42)	4 (7)	12 (34)	3 (5)	13 (30)	5 (6)	45 (38)	33 (14)	
Infants born alive	4	29	21	52	29	53	40	77	94	211	
Surfactant within 2 h of birth^e^											
Yes	0	5 (17)	12 (71)	42 (81)	20 (80)	45 (85)	13 (39)	65 (84)	45 (60)	157 (74)	0.02
No	0	24 (83)	5 (29)	10 (19)	5 (20)	8 (15)	20 (61)	12 (16)	30 (40)	54 (26)	
Intubation at birth^f^											
Yes	0	7 (24)	13 (81)	42 (81)	21 (78)	44 (83)	22 (55)	59 (77)	56 (68)	152 (72)	0.44
No	0	22 (76)	3 (19)	10 (19)	6 (22)	9 (17)	18 (45)	18 (23)	27 (32)	59 (28)	
Admission to NICU^g^											
Yes	1 (25)	7 (24)	16 (76)	45 (87)	23 (85)	52 (98)	40 (100)	77 (100)	80 (87)	181 (86)	0.79
No	3 (75)	22 (76)	5 (24)	7 (13)	4 (15)	1 (2)	0	0	12 (13)	30 (14)	

Abbreviation: NICU, neonatal intensive care unit.

^a^ Data missing for 9 (8%) observations in the 2004–2007 group.

^b^ Only for the subgroup of preterm labor.

^c^ Data missing for 11 (9%) observations in the 2004–2007 group and 3 (1%) observations in the 2012–2016 group.

^d^ Data missing for 2 (1%) patients in the 2012–2016 group.

^e^ Data missing for 19 (20%) observations in the 2004–2007 group.

^f^ Data missing for 11 (12%) observations in the 2004–2007 group.

^g^ Data missing for 2 (2%) observations in the 2004–2007 group.

Median birthweight was lower, and the rate of small for gestational age higher in infants born 2012–2016 (Table 3). The proportion of newborns with Apgar score ≤3 at 5 min decreased with increasing gestational week in both study groups. The rates were higher during 2012–2016 compared to during 2007–2004, but the differences were not statistically significant.

**TABLE 3:** Characteristics of liveborn infants. Values are numbers (percentages) unless stated otherwise.

	Week 22		Week 23		Week 24		Week 25		Week 22–25		
	2004–2007	2012–2016	2004–2007	2012–2016	2004–2007	2012–2016	2004–2007	2012–2016	2004–2007	2012–2016	*p*‐value
Number of infants	4	29	21	52	29	53	40	77	94	211	
Male	4 (100)	18 (62)	11 (52)	30 (58)	15 (52)	39 (74)	26 (65)	40 (52)	56 (60)	127 (60)	0.92
Female	0	11 (38)	10 (48)	22 (42)	14 (48)	14 (26)	14 (35)	37 (48)	38 (40)	84 (40)	
Birthweight											
Median range, g	470 (420–570)	480 (275–604)	600 (455–738)	585 (300–874)	674 (474–1070)	663 (429–876)	823 (266–1014)	761 (520–987)	700 (266–1070)	638 (275–987)	0.004
SGA^a^	0	3 (10)	0	4 (8)	2 (7)	9 (17)	4 (10)	16 (21)	6 (6)	32 (15)	0.003
Missing	0	0	0	1 (2)	2 (7)	0	0	1 (1)	2 (2)	2 (1)	
Apgar score at 5 min^b^											
≤3	4 (100)	26 (93)	7 (33)	18 (35)	10 (34)	21 (40)	2 (5)	12 (16)	23 (24)	77 (37)	0.04
4–10	0	2 (7)	14 (67)	34 (65)	19 (66)	32 (60)	38 (95)	65 (84)	71 (76)	133 (63)	
Number in birth											
Singleton	2 (50)	18 (62)	17 (81)	39 (75)	21 (72)	44 (83)	35 (88)	51 (66)	75 (80)	152 (72)	0.15
Multiples	2 (50)	11 (38)	4 (19)	13 (25)	8 (28)	9 (17)	5 (12)	26 (34)	19 (20)	59 (28)	

^a^ SGA, small for gestational age; birthweight of more than 2 standard deviations below the mean.

^b^ Data missing for 1 (0.5%) infant in the 2012–2016 group.

**FIGURE 1:**
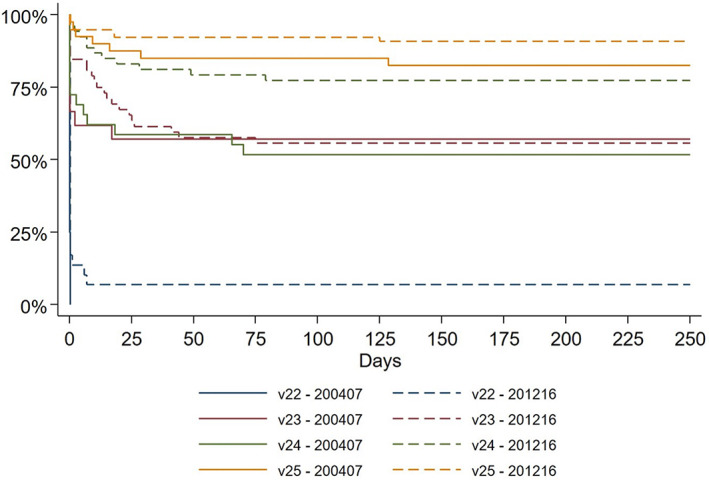
Survival to 1 year in liveborn infants according to gestational age.

Fetal and infant mortality and survival rates are presented in Table 4. Although the results indicate improvements between 2004–2007 and 2012–2016, most differences were non‐significant. Neither 1‐year survival nor 1‐year survival without major neonatal morbidity among liveborn infants changed significantly between the study periods. The rate of antepartum stillbirths decreased significantly, resulting in a higher proportion of infants born alive during 2012–2016 (88% vs. 80%), most obvious at 22–23 GW. More infants at 23–24 GW were admitted to NICU during 2012–2016, that is, the rate of delivery room deaths at these weeks decreased. Most deaths within 1‐year among liveborn infants occurred within 24 h and very few after the neonatal period (Figure 1).

**TABLE 4:** Mortality and survival rates, all infants. Values are numbers (percentages) unless stated otherwise.

	Week 22		Week 23		Week 24		Week 25		Week 22–25			
	2004–2007	2012–2016	2004–2007	2012–2016	2004–2007	2012–2016	2004–2007	2012–2016	2004–2007	2012–2016	*p*‐value*	Risk difference (95% CI)
All infants	9	44	31	57	35	58	43	81	118	240		
Stillborn (antepartum)	2 (22)	1 (2)	5 (16)	3 (5)	6 (17)	4 (7)	2 (5)	4 (5)	15 (13)	12 (5)	0.009	−7.7% (−14.3; −1.1)
Stillborn (intrapartum)	3 (33)	14 (32)	5 (16)	2 (4)	0	1 (2)	1 (2)	0	9 (8)	17 (7)	0.85	−0.5% (−6.3; 5.2)
Liveborn	4 (45)	29 (66)	21 (68)	52 (91)	29 (83)	53 (91)	40 (93)	77 (95)	94 (80)	211 (88)	0.04	8.3% (−0.1; 16.6)
Liveborn infants												
Admitted to NICU	1 (25)	7 (24)	16 (76)	45 (87)	23 (79)	52 (98)	40 (100)	77 (100)	80 (85)	181 (86)	0.88	0.7% (−7.9; 9.3)
Death <24 h	4 (100)	24 (83)	7 (33)	8 (15)	9 (31)	2 (4)	1 (3)	4 (5)	21 (22)	38 (18)	0.38	−4.3% (−14.2; 5.6)
Neonatal death 1–27 days	0	3 (10)	2 (10)	12 (23)	4 (14)	7 (13)	4 (10)	2 (3)	10 (11)	24 (11)	0.85	0.7% (−6.8; 8.3)
Death 28–365 days	0	0	0	3 (6)	1 (3)	3 (6)	2 (5)	1 (1)	3 (3)	7 (3)	1.00	0.1% (−4.2; 4.4)
Survival at 1 year												
Of all infants including stillbirths	0	2 (5)	12 (39)	29 (51)	15 (43)	41 (71)	33 (77)	70 (86)	60 (51)	142 (59)	0.14	8.3% (−2.6; 19.3)
Of liveborn infants	0	2 (7)	12 (57)	29 (56)	15 (52)	41 (77)	33 (83)	70 (91)	60 (64)	142 (67)	0.55	3.5% (−8.1; 15.1)
Of liveborns without major neonatal morbidity	0	0	2 (10)	6 (12)	3 (10)	8 (15)	15 (38)	30 (39)	20 (21)	44 (21)	0.93	−0.4% (−10.3; 9.5)

Abbreviations: CI, confidence interval; NICU, neonatal intensive care unit.

The proportion of 1‐year survivors with retinopathy of prematurity >grade 2 decreased between the study periods (55% to 39%); however the difference did not reach statistical significance. There were no differences in the proportions with intraventricular hemorrhage > grade 2, severe bronchopulmonary dysplasia periventricular leukomalacia, or necrotizing enterocolitis with or without surgery. Major morbidity by week is presented in Table S1.

Low gestational age at birth and low Apgar score at 5 min were the strongest independent factors associated with death within 1 year among infants born alive 2012–2016 (Table 5). Antenatal steroids, delivery by cesarean section and attendance of a neonatologist were protective factors in the univariable but not in the multivariable model.

**TABLE 5:** Risk factors of death within 1 year in infants born alive 2012–2016.

Variable	Number of infants	Number of events (%)	Univariable model		Multivariable model	
			Odds ratio (95% CI)	*p*‐value	Odds ratio (95% CI)	*p*‐value
Gestational age						
Week 22	29	27 (93)	135.00 (26.37–691.07)	<0.001	47.02 (3.67–602.12)	0.003
Week 23	52	23 (44)	7.93 (3.07–20.51)	<0.001	16.25 (3.75–70.30)	<0.001
Week 24	53	12 (23)	2.93 (1.07–8.03)	0.037	3.41 (0.79–14.71)	0.10
Week 25 (reference)	77	7 (9)	1.00	‐	1.00	‐
Maternal age						
<35 y (reference)	143	50 (35)	1.00	‐	1.00	‐
≥35 y	68	19 (28)	0.72 (0.38–1.36)	0.31	0.64 (0.23–1.78)	0.39
Maternal BMI						
<30 (reference)	165	49 (30)	1.00	‐	1.00	‐
≥30	38	17 (45)	1.92 (0.93–3.94)	0.077	2.91 (0.83–10.20)	0.095
Missing	8	3 (38)	1.42 (0.33–6.18)	0.64	0.63 (0.04–8.99)	0.73
Antenatal steroids						
Yes, any	185	48 (26)	0.08 (0.03–0.23)	<0.001	1.15 (0.09–15.54)	0.92
No (reference)	26	21 (81)	1.00	‐	1.00	‐
Infant sex						
Male	127	44 (35)	1.25 (0.69–2.27)	0.46	0.95 (0.37–2.46)	0.92
Female (reference)	84	25 (30)	1.00	‐	1.00	‐
Maternal antibiotics						
Yes	112	42 (38)	1.62 (0.89–2.92)	0.11	0.87 (0.28–2.74)	0.81
No (reference)	96	26 (27)	1.00	‐	1.00	‐
Missing	3	1 (33)	1.35 (0.12–15.48)	0.81	1.60 (0.03–83.11)	0.81
Pre‐eclampsia or hypertension						
Yes	17	4 (24)	0.61 (0.19–1.95)	0.40	1.03 (0.11–10.08)	0.98
No (reference)	194	65 (34)	1.00	‐	1.00	‐
PPROM						
Yes	52	22 (42)	1.77 (0.93–3.39)	0.085	2.39 (0.80–7.13)	0.12
No (reference)	157	46 (29)	1.00	‐	1.00	‐
Missing	2	1 (50)	2.41 (0.15–39.40)	0.54	6.62 (0.03–1279.77)	0.48
Chorioamnionitis or fever						
Yes	55	19 (35)	1.12 (0.58–2.14)	0.73	0.55 (0.17–1.76)	0.32
No (reference)	156	50 (32)	1.00	‐	1.00	‐
Placental abruption						
Yes	27	4 (15)	0.32 (0.11–0.96)	0.042	1.13 (0.24–5.32)	0.88
No (reference)	184	65 (35)	1.00	‐	1.00	‐
Number in birth						
Singleton (reference)	152	44 (29)	1.00	‐	1.00	‐
Multiple	59	25 (42)	1.80 (0.97–3.37)	0.064	2.51 (0.78–8.08)	0.12
Mode of delivery						
Vaginal birth	102	56 (55)	8.99 (4.47–18.07)	<0.001	2.15 (0.71–6.55)	0.18
Cesarean delivery (reference)	109	13 (12)	1.00	‐	1.00	‐
Birthweight						
SGA^a^	32	12 (38)	1.32 (0.60–2.89)	0.49	1.69 (0.32–8.93)	0.54
AGA^a^ (reference)	176	55 (3)	1.00	‐	1.00	‐
LGA^a^	1	0 (0)	‐	‐	‐	‐
Missing	2	2 (100)	‐	‐	‐	‐
Apgar score at 5 min						
≤3	77	51 (66)	13.38 (6.68–26.80)	<0.001	10.11 (3.55–28.78)	<0.001
4–10 (reference)	133	17 (13)	1.00	‐	1.00	‐
Missing	1	1 (100)	‐	‐	‐	‐
Neonatologist present						
Yes	198	57 (29)	0.04 (0.01–0.32)	0.002	0.19 (0.00–11.99)	0.44
No (reference)	11	10 (91)	1.00	‐	1.00	‐
Missing	2	2 (100)	‐	‐	‐	‐

Abbreviations: BMI, body mass index; CI, confidence interval; PPROM, preterm premature rupture of the membranes.

^a^ SGA, small for gestational age; birthweight of more than 2 standard deviations below the mean; AGA, appropriate for gestational age, birthweight–2 to 2 standard deviations of the mean; LGA, large for gestational age, birthweight more than 2 standard deviations over the mean.

## 4 DISCUSSION

In this single center cohort study at a tertiary university hospital, we could demonstrate significant changes in perinatal management between 2004–2007 and 2012–2016, with increased use of both obstetric and neonatal interventions in the later study period. Stillborn rate among fetuses alive at maternal admission decreased and the proportion of infants born alive increased in all gestational weeks; however, overall 1‐year survival and 1‐year survival without severe neonatal morbidity did not improve significantly between the study periods.

Perinatal management and survival rates among infants born extremely preterm, especially at 22–24 GW, vary considerably between and within high‐income countries and is largely a result of differences in attitudes and guidelines.^22^, ^23^, ^24^, ^25^ Regional and Swedish guidelines from 2013–2016 recommend an increasingly proactive approach.^13^, ^14^ This study confirms that Karolinska University Hospital Solna largely has accommodated to these guidelines. This is also reflected by the almost four‐fold increase in livebirths at 22 GW between the study periods, caused by a higher transfer rate (during 2004–2007 20% of liveborns at 22 GW in the Stockholm region were born at Karolinska, compared to 83% during 2012–2016; data shown upon request). Obstetric and neonatal intervention rates at 23–25 GW during 2012–2016 were comparable to those in Sweden overall, as presented by Norman et al. in the EXPRESS follow‐up study.^2^ However, at 22 GW, intervention rates were clearly lower and low compared to those in the EXPRESS follow‐up study. For example, at this week, antenatal steroids were administered in 23% during 2012–2016 in our study compared to 64% in the EXPRESS follow‐up study, and a neonatologist was attending in 50% in our study compared to 90% in the EXPRESS follow‐up study. An explanation might be that the present study included births from 2012–2013, that is, 2 years earlier than the EXPRESS follow‐up study, and although a shift toward more active perinatal management was ongoing, this was from a very low level and at these years before the national guidelines were published. Most guidelines in other high‐income countries at the same time supported comfort care at 22 GW which might have influenced the attitudes.^26^ To note, in the EXPRESS follow‐up study, only liveborn infants were included when calculating intervention rates while in this study all infants were included, resulting in slightly lower rates. Cesarean delivery rates increased three‐fold between 2004–2007 and 2012–2016, despite scarce evidence for the benefit. A Cochrane review from 2012, including only 116 women, could not confirm the benefit of cesarean delivery of preterm singletons.^27^ In contrast, a recent large meta‐analysis of observational data comparing delivery mode in vertex infants <28 GW indicated decreased adjusted odds ratios of infant death with cesarean delivery.^28^ Due to weak evidence for the benefits and concerns about complication risks, Swedish guidelines recommend cesarean delivery at first from 23–24 GW, and thus, this was rare at 22 GW during both study periods.

Despite the increased proportion of liveborn infants between the study periods, we could not demonstrate a significant increase in 1‐year survival among liveborn infants; 64% vs 67%. Due to the low number of infants, large changes in outcomes over time would have been required to prove statistical significance and thus comparisons with other studies must be done with caution. In the EXPRESS follow‐up study from 2014–2016, 1‐year survival increased significantly from 64% to 71% for births 22–25 GW (births at 26 GW excluded).^2^ Our less favorable 1‐year survival 2012–2016 was almost entirely restricted to week 22, at which only 7% of liveborn infants survived to 1 year compared to 30% in the EXPRESS follow‐up study. This probably reflects the lower perinatal activity in this week. The 22‐week survival is, however, comparable with reports from other; 7.3% in a meta‐analysis of studies in high‐income countries published 2000–2017, and 11% in a US study from 2013–2018 by Bell et al.^1^, ^29^ Importantly, although 1‐year survival did not increase between the study periods, neither did it decrease, despite a significantly higher proportion of infants born at 22–23 GW during 2012–2016 (38% vs. 26% 2004–2007), indicating more active and improved management at the border of viability.

In the present study, 1‐year survival without major neonatal morbidity was 21% during both study periods. This outcome may naturally be more difficult to improve; with more active perinatal management more infants will survive, but fragile and prone to complications. A lower median birthweight and more small‐for‐gestational‐age infants during 2012–2016 in our study support such an assumption. In comparison, the EXPRESS follow‐up survival without major morbidity for infants born alive at 22–25 GW increased from 23% 2004–2007 to 29% 2012–2016, and in a Canadian study, it improved from 7% to 12.9% among infants born at 23–25 GW, including sepsis in the definition of major morbidity.^2^, ^4^

Low Apgar score at 5 min was a predictor of death within 1 year in this study. A large nationwide study in Sweden found the same association across all gestational ages <37 GW; however, contradictory findings have been published.^21^, ^30^, ^31^ To initiate resuscitation and NICU‐care with continuous reconsideration is proposed to be a reasonable and ethical way to handle newborns at the border of viability when infant status may not give prognostic information.^32^

Our results are essentially in line with those in other studies from the same time and support the adherence to current guidelines on perinatal management of extremely preterm births. Continued adaptation to the recommendation to consider active care at 22 GW is reasonable.

The findings underline the importance of regular evaluations of the care at individual centers, to relate to guidelines and results from others, and to inform parents‐to‐be.

A strength of this study was the inclusion of all births at one single level III center during both study periods. Definitions of variables were the same across the study periods and all outcome diagnoses were crosschecked and validated against relevant registers and medical records. Several limitations must be addressed. The main limitation was the low number of infants making the study underpowered to detect small changes over time. Data were collected slightly differently for the study periods; during 2004–2007 prospectively as part of the EXPRESS‐study and validated against registers vs during 2012–2016 from medical records and registers and validated against medical records. Further, it cannot be ruled out that morbidity diagnoses may have been applied slightly differently across the study periods. Both severe bronchopulmonary dysplasia and necrotizing enterocolitis have had slightly different diagnostic criteria over time and in different settings. A potential overestimation of necrotizing enterocolitis was discussed in the EXPRESS follow‐up study.^2^ Lastly, the study did not include neurodevelopmental follow‐up data.

## 5 CONCLUSION

At this tertiary university hospital in Sweden, overall rates of obstetric and neonatal interventions at births 22–25 GW increased substantially between 2004–2007 and 2012–2016; however, at 22 GW, they were still at a low level during 2012–2016. One‐year survival among liveborn infants did not change despite a higher proportion of those born at 22–23 GW.

## AUTHOR CONTRIBUTIONS

Study concept and planning: EA and SS; Data collection: EA, SS, MB; Analysis and interpretation of findings: all authors; Manuscript writing; all authors.

## CONFLICT OF INTEREST STATEMENT

None.

## Supporting information


Table S1.
